# Left atrial stiffness as a novel echocardiographic parameter of atrial dysfunction in cats with hypertrophic cardiomyopathy: A two-dimensional speckle-tracking study

**DOI:** 10.14202/vetworld.2025.4046-4055

**Published:** 2025-12-23

**Authors:** Patara Tohthong, Jidapa Tosuwan, Sirilak Disatian Surachetpong

**Affiliations:** 1 Graduate Program in Veterinary Medicine, Faculty of Veterinary Science, Chulalongkorn University, Bangkok, 10330, Thailand; 2 Department of Veterinary Medicine, Faculty of Veterinary Science, Chulalongkorn University, Bangkok, 10330, Thailand

**Keywords:** diastolic dysfunction, feline cardiology, hypertrophic cardiomyopathy, left atrial function, left atrial stiffness, speckle-tracking echocardiography

## Abstract

**Background and Aim:**

Hypertrophic cardiomyopathy (HCM) is the most common form of cardiomyopathy in cats and is often linked with diastolic dysfunction and progressive remodeling of the left atrium (LA). While LA strain analysis has been utilized to measure atrial function, LA stiffness (LASt), a load-adjusted marker that combines diastolic filling pressures and atrial deformation (E:E’/εS), has not yet been studied in feline patients. This study aimed to compare LASt between normal cats and those with HCM and to assess its correlation with traditional echocardiographic parameters of LA and left ventricular (LV) function.

**Materials and Methods:**

This retrospective cross-sectional study included client-owned cats evaluated at a university teaching hospital between August 2021 and August 2022. Cats were classified as normal or HCM based on LV wall thickness (≤5 mm vs. ≥6 mm). Standard echocardiographic parameters, doppler indices, tissue Doppler imaging (TDI) values, and two-dimensional speckle-tracking echocardiography (STE)–derived LA strain variables (reservoir, conduit, and active strain) were measured. LASt was calculated as E:E’/εS. Group comparisons were performed using t-tests or Mann–Whitney U tests, and correlations were assessed using Pearson’s coefficient.

**Results:**

Thirty-seven cats met the inclusion criteria (12 with HCM and 25 normal). Cats with HCM showed significantly higher LASt values compared to normal cats (median 2.26 vs. 0.30; p < 0.001), representing approximately a sevenfold increase. Reservoir strain (εS), conduit strain (εE), and active strain (εA) were all significantly lower in the HCM group (*p* < 0.001). LASt showed strong positive correlations with LA diameter and LA:Ao ratio (r ≥ 0.85; p < 0.001), and moderate-to-strong correlations with Doppler and TDI markers of diastolic dysfunction, including E:A ratio, MV A velocity, MV E’, and MV S’.

**Conclusion:**

LASt, measured by STE, provides a sensitive, load-inclusive index of LA mechanical dysfunction in cats with HCM and may detect atrial remodeling earlier than strain alone. Its strong association with established markers of diastolic impairment supports its potential clinical utility for identifying subclinical atrial dysfunction. Larger longitudinal studies are warranted to validate LASt as a prognostic biomarker and to define clinically relevant thresholds for disease staging and monitoring.

## INTRODUCTION

Hypertrophic cardiomyopathy (HCM) is a common heart condition in cats, with an estimated prevalence of up to 15% in the general feline population [1]. It is the most frequently diagnosed form of cardiomyopathy and is characterized by concentric left ventricular (LV) hypertrophy with typically preserved systolic function [2]. In some cats, dynamic LV outflow tract obstruction may develop due to systolic anterior motion of the septal mitral valve leaflet, leading to increased filling pressures, decreased coronary perfusion, and progression of myocardial fibrosis [3]. HCM can occur at any age, and cardiovascular disease is one of the leading causes of death in cats [4].

Diastolic dysfunction is widely acknowledged as the primary pathophysiological feature driving HCM progression. Impaired LV relaxation increases ventricular filling pressures, making affected cats more prone to pulmonary congestion, pulmonary edema, and secondary atrial enlargement [3]. Diagnosis can be challenging because clinical signs may be subtle or absent until severe complications such as arterial thromboembolism (ATE) or congestive heart failure (CHF) develop [4]. Currently, combining cardiac biomarkers, especially N-terminal pro–brain natriuretic peptide (NT-proBNP), with echocardiography offers an effective method for early screening and clinical assessment of HCM [5].

Echocardiography allows for direct assessment of LV wall thickness and disease staging, although most parameters mainly focus on LV structure and function. Only certain indices, such as LA size, the LA:AO ratio, and LA fractional shortening (LAFS), are routinely used to evaluate left atrium (LA) structure and performance. Despite its essential role in modulating LV filling and preventing CHF, LA function is often underestimated due to its complex physiology [3]. The LA acts as a reservoir during systole, a conduit during early diastole, and a booster pump during late diastole, and these phases can be evaluated through echocardiography [6].

LA reservoir strain (εS), also called peak atrial longitudinal strain, has proven useful to assess LA function in feline HCM [7]. In human cardiology, LA function is evaluated through volumetric analysis, pulmonary vein flow, transmitral doppler patterns, tissue Doppler imaging (TDI), and deformation imaging such as two-dimensional speckle-tracking echocardiography (STE) [6]. Increased left atrial stiffness (LASt), calculated as E:E’/εS [8, 9], has become a strong prognostic indicator in humans with HCM, predicting outcomes like CHF, hospitalization, and sudden cardiac death, especially in those with LV outflow tract obstruction [10]. Early detection of abnormal LA mechanics using LASt may improve clinical decision-making, prognosis, and allow earlier therapeutic intervention.

Although LA strain indices (εS, εE, and εA) have been used to characterize atrial dysfunction in cats with HCM [7, 11], atrial stiffness has not yet been evaluated by integrating strain with indices of diastolic load. Unlike conventional strain, which depends on preload, LASt combines both loading conditions and myocardial deformation, offering a more comprehensive measure of atrial compliance.

Although left atrial strain parameters (εS, εE, and εA) are increasingly used to evaluate atrial mechanical function in cats with HCM, current methods are limited by their reliance on loading conditions and inability to quantify the interaction between atrial deformation and diastolic filling pressures. In contrast, left atrial stiffness (LASt), which combines strain with Doppler-derived indices of ventricular filling (E:E’), has proven to be a sensitive and prognostic marker in humans with HCM and other diastolic heart diseases. Despite its clinical significance in human cardiology and its emerging relevance in canine cardiology, LASt has never been studied in feline patients. No research has assessed whether LASt can distinguish normal cats from those with HCM, detect early atrial remodeling, or reflect the severity of diastolic dysfunction. Furthermore, the relationship between LASt and established echocardiographic indicators, including LA size, LAFS, strain components, and Doppler/TDI parameters, remains unknown in cats. This lack of species-specific data creates a critical gap, considering the high prevalence of HCM in cats, the difficulties in early diagnosis, and the need for reliable, noninvasive markers of atrial compliance.

This study aimed to introduce and evaluate LASt as a novel echocardiographic parameter for assessing atrial compliance in cats with HCM. Specifically, the study sought to (i) compare LASt values between healthy cats and those diagnosed with HCM using two-dimensional speckle-tracking echocardiography (STE) and Doppler-derived diastolic indices, and (ii) examine the correlations between LASt and traditional echocardiographic measures, including LA size, LA:AO ratio, strain parameters, transmitral flow velocities, and tissue doppler indices. By combining functional deformation data with diastolic loading conditions, this study aims to determine whether LASt offers a more sensitive and comprehensive marker of atrial dysfunction than strain alone. The overall goal is to assess the potential of LASt as an early diagnostic tool and future prognostic indicator in feline HCM.

## MATERIALS AND METHODS

### Ethical approval

This study was conducted in accordance with the ethical principles and guidelines for the care and use of animals in research. Ethical approval for the use of clinical data and echocardiographic images of client-owned cats was obtained from the Institutional Animal Care and Use Committee of the Faculty of Veterinary Science, Chulalongkorn University, Thailand (Approval No. 2431052/2021). All procedures complied with the institutional regulations, the National Research Council of Thailand’s guidelines for animal research, and the Code of Practice for the Care and Use of Animals for Scientific Purposes.

Because this was a retrospective cross-sectional study, no experimental interventions, sedations, or invasive procedures were performed for research purposes. Only previously acquired clinical echocardiographic images, ultrasonographic cine loops, physical examination findings, and diagnostic measurements were used. All cats were presented for routine clinical evaluation at the Cardiology Clinic of the Small Animal Teaching Hospital, and data were included only after obtaining written informed consent from the owners allowing the use of anonymized medical records for research. Confidentiality of owner and patient information was strictly maintained.

The study involved no additional handling, no alteration of standard diagnostic protocols, and no procedures beyond routine veterinary care. All echocardiographic examinations in the medical records were performed by a licensed veterinarian following American College of Veterinary Internal Medicine (ACVIM) guidelines for cardiac imaging, without sedation unless clinically indicated. No animal was harmed or subjected to any stress, pain, or risk for the purpose of the study. The use of client-owned animals followed the principles of Replacement, Reduction, and Refinement (the 3Rs), ensuring that only necessary and high-quality datasets were included to minimize any unnecessary use of animals.

### Study period and location

The study was conducted from August 2021 and August 2022 at the Cardiology Clinic, Small Animal Teaching Hospital, Faculty of Veterinary Science, Chulalongkorn University, Thailand.

### Study design

This retrospective cross-sectional study. The required sample size was determined using G*Power 3.1.9.7 software. Based on the mean and standard deviation (SD) reported in a previous study on LASt in dogs with myxomatous mitral valve disease [8], an effect size of 0.7964 was identified. With a Type I error of 0.05 and a power of 0.95, the minimum sample size was calculated to be 8 cats per group.

### Animal selection

Clinical and echocardiographic data were collected from the Chulalongkorn Hospital Information System and the records of cats that visited the Cardiology Clinic during the study period. Cats were divided into two groups:

- HCM group: Cats with LV wall thickness ≥6 mm in diastole.
- Normal group: Cats with LV wall thickness ≤5 mm and no evidence of systemic or cardiac disease.

Cats were excluded if they had borderline LV wall thickness (5–6 mm), systemic hypertension (systolic blood pressure [SBP] >160 mmHg), hyperthyroidism (total T4 >4 μg/dL), arrhythmias, congenital heart defects, or any systemic disorder. Only echocardiographic studies with clear endocardial border visualization throughout the cardiac cycle were included.

All cats underwent complete physical examinations, blood tests, doppler blood pressure measurements, and electrocardiography before or on the same day as echocardiographic evaluation. Cats in the HCM group that experienced CHF were treated with medications such as furosemide, clopidogrel, or pimobendan prior to echocardiography.

### Echocardiography

Echocardiographic examinations were conducted using a Mindray M9 ultrasound system (Shenzhen, China) equipped with a 4–10 MHz phased-array transducer and a Tissue Tracking Quantitative Analysis module. Cats were scanned without sedation in right and left lateral recumbency. Standard echocardiographic views were obtained following ACVIM guidelines. All examinations were performed by a single investigator (SS) to reduce inter-observer variability.

M-mode and two-dimensional (2D) images were used to measure:

- LV internal diameter in diastole (LVIDd) and systole (LVIDs)
- Interventricular septal thickness (IVSd, IVSs)
- LV posterior wall thickness (LVPWd, LVPWs)
- LA and aortic (AO) diameter

LV fractional shortening (FS) was calculated as:

FS = [(LVIDd – LVIDs)/LVIDd] × 100

The LA:AO ratio was derived from 2D images. Left atrial diameters at end-diastole (LAD) and end-systole (LAS) were measured using M-mode. Left atrial FS was calculated as [12]:

LAFS = [(LAD – LAS)/LAD] × 100

Pulsed-wave doppler from the left apical four-chamber view was used to measure early (E) and late (A) transmitral flow velocities and calculate the E:A ratio. Isovolumic relaxation time (IVRT) was measured from the apical five-chamber view [13]. Pulmonary venous flow (systolic, diastolic, reversal waves) was assessed from the right parasternal short-axis view [4, 12–14].

Lateral mitral annular velocities were measured from the left apical four-chamber view, including systolic (S’), early diastolic (E’), and late diastolic (A’) velocities [15].

Left atrial myocardial deformation was assessed using 2D speckle-tracking from left apical four-chamber cine loops acquired at ≥60 frames per second. The LA endocardial border was manually traced at end-systole, and the region of interest was automatically adjusted to encompass the entire myocardial thickness.

Global LA strain components were calculated as:

- Reservoir strain (εS) – ventricular systole
- Conduit strain (εE) – early diastole
- Active strain (εA) – late diastole

Left atrial stiffness (LASt) was calculated as [8]:

LASt = E:E’ / εS

Where E = early transmitral flow velocity and E’ = early diastolic mitral annular velocity.

**Figure 1:**
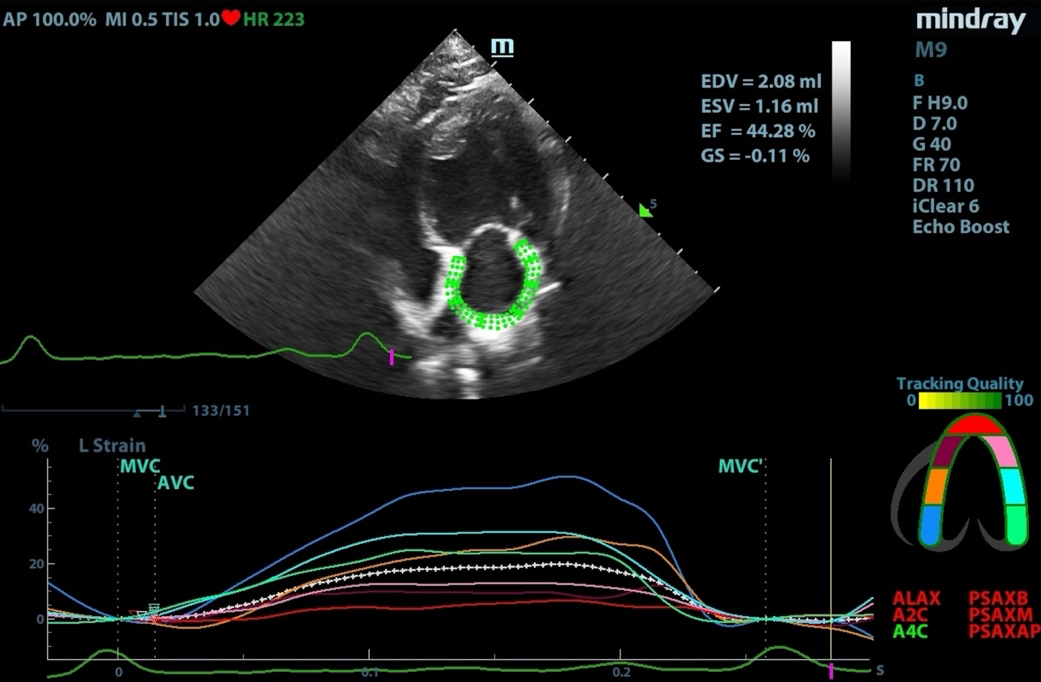
Left atrial strain assessed using two-dimensional speckle-tracking. The colored lines indicate strain in each segment of the left atrial wall, while the dotted line represents the global strain of the left atrium.

Strain analyses were performed by a single blinded investigator (PT) (Figure 1). Intra- and interobserver coefficients of variation for εS were <15%, consistent with previous findings [11].

### Statistical analysis

Data were analyzed using IBM SPSS Statistics version 29.0.1 (IBM Corp., Armonk, NY, USA). Normality was tested with the Shapiro–Wilk test.

- Normally distributed variables are presented as mean ± SD.
- Non-normally distributed variables are expressed as median interquartile range (IQR).

Between-group comparisons were conducted using the Student’s t-test for parametric data and the Mann–Whitney U test for non-parametric data. Correlations between LASt and echocardiographic parameters were assessed using Pearson’s correlation coefficient, with significance set at p < 0.05. Correlation strength was categorized according to Chan [16], with r ≥ 0.8 considered strong, 0.6–0.8 moderate, 0.3–0.5 fair, and < 0.3 weak.

## RESULTS

### Study population

Data from 38 cats were initially collected; however, one cat was excluded due to incomplete records. A total of 37 cats met the inclusion criteria, including 12 with HCM and 25 normal cats. The study population included a variety of breeds, such as Scottish Fold, Domestic Shorthair, Persian, Maine Coon, mixed breeds, American Shorthair, Bengal, and Himalayan.

Sex distribution varied between groups. The HCM group was mainly males (11 males, 91.7%; 1 female, 8.3%), while the normal group had 16 males (64%) and 9 females (36%). Table 1 outlines the baseline characteristics. Among all baseline variables, only the vertebral heart score (VHS) was significantly higher in the HCM group compared to normal cats. No significant differences were observed in age, body weight, SBP, or heart rate (HR) between the groups. Cats diagnosed with HCM received various cardiac medications prior to imaging, including furosemide (n = 6), enoxaparin (n = 9), and clopidogrel (n = 5). None received pimobendan.

**Table 1:** Signaling and baseline characteristics of cats with and without HCM.

Variables	HCM (n = 12)	Normal (n = 25)	p-value
Age (years)	4 (3, 8)	4 (1, 6)	0.377
Body weight (kg)	4.8 (4.48, 5.23)	4.2 (3.4, 6.0)	0.236
VHS	8.4 (7.60, 9.05)	7.3 (6.85, 7.45)	0.019*
SBP (mmHg)	145 (115, 150)	128.0 (118.0, 137.0)	0.335
HR (bpm)	200 (181, 225)	196.0 (184.5, 206.5)	0.419
Breeds			
Domestic Shorthair	8	6	
Scottish Fold	–	11	
Persian	1	3	
Maine Coon	1	2	
Mixed	2	–	
American Shorthair	–	1	
Bengal	–	1	
Himalayan	–	1	

Data are presented as median interquartile range.

* Indicates statistical significance at a p-value of 0.05, analyzed using the Mann–Whitney *U* test. HCM = Hypertrophic cardiomyopathy, VHS = Vertebral heart score, SBP = Systolic blood pressure, HR = Heart rate.

### Conventional echocardiographic findings

Conventional echocardiographic measurements are reported as median (interquartile range) values and analyzed using the Mann–Whitney U test (Table 2). As expected, cats with HCM showed increased LV wall thicknesses, larger LA dimensions, and altered doppler flow parameters consistent with diastolic dysfunction, compared to normal cats.

**Table 2:** Conventional echocardiographic measurements in normal cats and cats with HCM.

Criteria	HCM (n = 12)	Normal (n = 25)	p-value
2-Dimensional measurement			
LA (cm)	1.53 (1.40, 2.00)	1.12 (1.02, 1.21)	0.0002*
AO (cm)	0.74 (0.68, 0.83)	0.85 (0.80, 0.99)	0.009*
LA:AO	2.19 (1.84, 2.98)	1.25 (1.15, 1.44)	<0.0001*
M-mode measurement			
IVSd (cm)	0.62 (0.43, 0.82)	0.45 (0.40, 0.50)	0.019*
IVSs (cm)	0.90 (0.81, 0.98)	0.69 (0.63, 0.76)	0.0005*
LVIDd (cm)	1.36 (1.08, 1.67)	1.41 (1.31, 1,47)	0.81
LVIDs (cm)	0.78 (0.42, 0.90)	0.57 (0.44, 0.73)	0.432
LVPWd (cm)	0.62 (0.60, 0.67)	0.43 (0.38, 0.46)	<0.0001*
LVPWs (cm)	0.85 (0.77, 0.96)	0.71 (0.63, 0.80)	0.003*
FS (%)	48.28 (39.89, 57.82)	56.05 (50.17, 66.25)	0.061
Spectral doppler measurements			
MV E vel (cm/s)	70.15 (47.03, 82.02)	75.38 (63.95, 86.75)	0.215
MV A vel (cm/s)	34.64 (28.56, 50.61)	59.62 (52.04, 68.38)	0.0005*
E:A	1.56 (1.32, 2.10)	1.19 (1.10, 1.40)	0.026*
IVRT (ms)	50 (40, 60)	45 (40, 50)	0.233
AV vel (cm/s)	81.04 (64.73, 106.56)	90.15 (83.23, 97.31)	0.532
PV vel (cm/s)	83.13 (59.98, 99.42)	86.39 (76.04, 92.10)	0.818
PV PG (mmHg)	2.77 (1.49, 3.98)	2.99 (2.31, 3.44)	0.759
Pvein S vel (cm/s)	38.42 (24.50, 44.18)	51.76 (39.27, 59.85)	0.019*
Pvein D vel (cm/s)	28.63 (23.86, 30.79)	40.70 (37.49, 46.71)	0.0005*
Even A vel (cm/s)	14.82 (13.40, 20.08)	15.35 (13.57, 19.06)	0.733
Even A dur (ms)	70 (50, 80)	46 (43, 56)	0.012*
Tissue doppler imaging (TDI)			
MV S’ vel (cm/s)	5.42 (5.10, 6.79)	8.92 (7.70, 10.53)	<0.0001*
MV E’ vel (cm/s)	6.96 (5.59, 8.15)	11.53 (10.58, 13.47)	0.0001*
MV A’ vel (cm/s)	5.51 (4.60, 7.43)	7.95 (6.51, 8.94)	0.002*
E’:A’	1.36 (0.81, 1.54)	1.47 (1.34, 1.77)	0.160
LA measurement			
LAD (cm)	1.96 (1.58, 2.28)	1.20 (1.08, 1.35)	0.0002*
LAS (cm)	1.68 (1.38, 2.12)	0.85 (0.78, 0.95)	0.0002*
LAFS (%)	10.42 (8.07, 16.61)	27.17 (33.99, 24.80)	0.0004*
LAA flow velocity (cm/s)	18.31 (16.34, 22.79)	34.60 (29.51, 40.42)	0.0009*

Data are presented as median interquartile range.

* Indicates statistical significance at a p-value of 0.05, analyzed using the Mann–Whitney U test. Ao = Aorta, AV vel = Peak velocity of aortic valve flow, E:A = Ratio of MV E to MV A, E’:A’ = Ratio of MV E’ to MV A’, FS = Left ventricular fractional shortening, HCM = Hypertrophic cardiomyopathy, IVRT = Isovolumic relaxation time, IVSd = Interventricular septal thickness at end-diastole, IVSs = Interventricular septal thickness at end-systole, LA = Left atrium, LA:Ao = Ratio of LA to AO, LAA flow vel = Left atrial appendage flow velocity, LAD = Left atrial size at LA diastole, LAS = Left atrial size at LA systole, LAFS = Left atrial fractional shortening, LV = Left ventricle, LVIDd = Left ventricular internal diameter at end-diastole, LVIDs = Left ventricular internal diameter at end-systole, LVPWd = Left ventricular posterior wall thickness at end-diastole, LVPWs = Left ventricular posterior wall thickness at end-systole, MV A = Peak velocity of late diastolic transmitral flow, MV A’ = Peak velocity of late diastolic mitral annular motion as determined by pulsed-wave doppler, MV E = Peak velocity of early diastolic transmitral flow, MV E’ = Peak velocity of early diastolic mitral annular motion as determined by pulsed-wave doppler, MV S’ = Peak velocity of systolic mitral annular motion as determined by pulse wave doppler, PV vel = Peak velocity of pulmonary valve flow, PV pg = Peak pressure gradient of pulmonary valve flow, Pvein A = Peak velocity of pulmonary vein reversal flow during atrial contraction, Pvein A dur = Duration of pulmonary vein flow reversal at atrial contraction, Pvein D = Peak velocity of diastolic pulmonary vein flow, Pvein S = Peak velocity of systolic pulmonary vein flow.

### STE-derived LA function and stiffness

LASt was measured using STE. STE-derived parameters, reservoir strain (εS), conduit strain (εE), active strain (εA), and LASt are listed in Table 3.

**Table 3:** Two-dimensional speckle-tracking echocardiography of normal and hypertrophic cardiomyopathy cats.

Variables	HCM (n=12)	Normal (n=25)	Median difference (95% CI)	p-value
E:E’	7.81 (6.53, 15.49)	6.50 (5.12, 7.35)	–1.892 (–4.80 to –0.41)	0.018*
εS (%)	3.18 (2.07, 4.83)	21.37 (18.70, 25.69)	17.66 (15.08–20.75)	<0.0001*
εE (%)	–1.49 (-1.81, -0.91)	–12.92 (–15.11, -9.96)	–10.93 (–13.05 to -9.09)	<0.0001*
εA (%)	–1.53 (-2.87, -0.94)	–8.62 (–11.75, -6.40)	–7.18 (–8.93 to –4.96)	<0.0001*
LASt	2.26 (1.45, 4.95)	0.30 (0.26, 0.37)	–1.96 (–4.29 to –1.40)	<0.0001*

Data are presented as median interquartile range.

* Indicates statistical significance at a p-value 0.05, analyzed using the Mann–Whitney U test. CI = Confidence interval, E:E’ = Ratio of MV E to MV E’, εA = Left atrial conduit strain, εE = Left atrial conduit strain, εS = Left atrial reservoir strain, LASt = Left atrial stiffness.

Key findings include:

- LASt was significantly higher in the HCM group than in normal cats, indicating markedly reduced atrial compliance.
- The E:E’ ratio was significantly increased in HCM cats, reflecting elevated LV filling pressures.
- εS was significantly lower in cats with HCM compared with normal cats.
- εE and εA were significantly less negative, further indicating impaired LA mechanical function in HCM.

### Correlation between LASt and echocardiographic parameters

**Figure 2:**
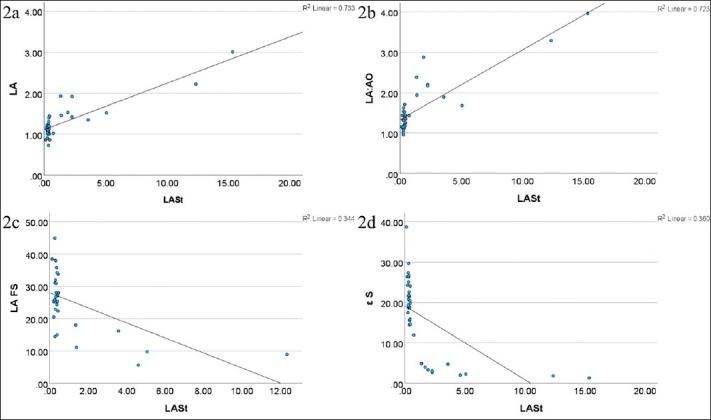
(a) Correlation between left atrial stiffness and left atrial size, (b) Left atrial-to-aorta ratio (LA:Ao), (c) Left atrial fractional shortening, and (d) Left atrial reservoir strain (εS).

Correlation analyses between LASt and conventional echocardiographic variables are summarized in Table 4. Major correlation trends include (Figure 2):

**Table 4:** Correlation between left atrial stiffness and other echocardiographic parameters.

Parameters	Pearson’s r	p-value
Characteristics		
Age (years)	–0.008	0.965
Body weight (kg)	0.060	0.723
HR (bpm)	0.257	0.124
Conventional echocardiography		
LA diameter (cm)	0.856*	<0.001
AO diameter (cm)	–0.263	0.126
LA:AO	0.850*	<0.001
MV E vel (cm/s)	0.169	0.318
MV A vel (cm/s)	–0.608*	<0.001
E:A	0.887*	<0.001
IVRT (ms)	–0.060	0.727
Pvein S vel (cm/s)	–0.185	0.286
Pvein D vel (cm/s)	–0.401*	0.017
Pvein A vel (cm/s)	–0.119	0.495
Pvein A dur (ms)	0.178	0.315
MV S’ (cm/s)	–0.536*	<0.001
MV E’ (cm/s)	–0.554*	<0.001
MV A’ (cm/s)	–0.180	0.286
E’:A’	–0.511*	0.001
LAD (cm)	0.719*	<0.001
LAS (cm)	0.752*	<0.001
LAFS (%)	–0.586*	<0.001
LAA flow velocity (cm/s)	–0.483*	0.007

E:E’	0.872*	<0.001
εS (%)	–0.600*	<0.001
εE (%)	0.501*	0.002
εA (%)	0.518*	0.001

- Strong positive correlations with LA diameter and LA:AO ratio, confirming the association between LASt and structural atrial remodeling.
- Moderately strong positive correlations with LAD and LAS, supporting its relationship with LA size.
- Fair negative correlations with FS, LAFS, and LAA flow velocity, indicating functional impairment with increasing stiffness.
- Positive correlations with strain components εE and εA.
- In doppler parameters, LASt showed a strong positive correlation with E:A and a moderately strong negative correlation with MV A.
- A fair negative correlation was noted with Pvein D velocity.

For TDI variables, LASt showed fair negative correlations with MV E’ and MV S’, and no significant correlation with MV A’.

## DISCUSSION

### Introduction of LASt as a novel marker in feline cardiology

This study is the first in feline cardiology to introduce LASt, a composite index derived from the ratio of transmitral flow velocity to myocardial deformation (E:E’/εS), as a new echocardiographic marker for assessing atrial compliance in cats with HCM. LASt values were significantly higher in cats with HCM, showing nearly a sevenfold increase compared to normal cats (median 2.26 vs. 0.30, p < 0.001). This notable increase indicates the presence of considerable atrial stiffening and structural remodeling. Although a diagnostic cutoff could not be determined due to a limited sample size, the results suggest that LASt has the potential to distinguish between healthy and diseased cats and to serve as a marker of disease progression. Additional STE-derived parameters—εS, εE, and εA, were also significantly impaired in HCM cats, further indicating reduced atrial mechanical function.

### Baseline characteristics and their implications

Both groups exhibited similar baseline characteristics regarding age, body weight, SBP, and HR. The VHS was the only variable significantly higher in the HCM group, indicating structural cardiac changes consistent with previous studies [17]. This supports the idea that LASt elevation in HCM cats is linked not to demographic factors but to disease-related myocardial alterations.

### Conventional echocardiographic and doppler findings

Conventional echocardiographic parameters confirmed expected structural differences between groups, including increased LA and LV sizes in cats with HCM. The significant decrease in MV A and the increase in E:A ratio suggest elevated LA pressure and impaired diastolic function [18]. TDI also showed reductions in MV S’, E’, and A’, indicating compromised systolic and diastolic annular motion. Changes in pulmonary venous flow and reduced LAA flow velocity further supported the presence of atrial dysfunction. Additionally, reductions in εS, εE, and εA verified previous research findings on LA strain abnormalities in HCM [11].

### Physiological basis and mechanistic interpretation of LASt

LASt offers an advantage over using strain parameters alone because εS depends on preload, and changes in loading conditions can influence strain measurements. LA fibrosis and decreased compliance restrict atrial deformation, reducing all strain components (εS, εE, and εA). By including the E:E’ ratio, LASt accounts for diastolic loading and delivers a more consistent and comprehensive evaluation of atrial stiffness [8].

The increased LASt observed in HCM likely indicates elevated LV filling pressures, which transmit backward to the LA, raising atrial pressure, wall stress, and the stimulus for atrial fibrotic remodeling [3, 19]. Such fibrosis decreases compliance, further increasing LASt, aligning with findings in human patients [20]. Elevated LASt may therefore contribute to pulmonary venous hypertension, congestion, and pulmonary edema [21]. Importantly, LASt may detect early atrial remodeling before obvious LA enlargement, identifying subclinical disease stages.

### Correlations of LASt with structural and functional parameters

LASt was independent of age, body weight, and HR, confirming its stability across physiological variations. Strong correlations with LA diameter, LA:Ao ratio, LAD, and LAS demonstrate its association with anatomical enlargement. Negative correlations with FS, LAFS, and LAA flow indicate functional impairment as stiffness increases. Associations with E:A, MV A, MV S’, and MV E’ suggest that LASt reflects the interaction between atrial and ventricular diastolic dynamics. Compared to εS, which only doubles in cats with HCM, LASt shows a sevenfold increase, highlighting its greater sensitivity for detecting atrial dysfunction. Additionally, LASt values in HCM cats were nearly three times higher than those reported in dogs with stage C MMVD [8], indicating species-specific differences and potential clinical thresholds.

### Study limitations

Several limitations must be recognized. Feline stress and increased HR during hospital visits might have affected doppler measurements, especially the E and A wave summation. The retrospective design led to occasional missing data and potential selection bias; however, only complete, high-quality datasets were included. The small number of HCM cats reflects the challenge of obtaining STE-optimized cine loops, as strain accuracy relies on frame rate and full visualization of the atrial wall. Pre-treatment with medications like furosemide may have changed preload and influenced E:E’ and strain values. Finally, correlation analyses used Pearson’s coefficient without adjusting for multiple comparisons, raising the risk of Type I errors. Larger, prospective longitudinal studies are necessary to validate LASt, define diagnostic cutoffs, and evaluate prognostic performance across HCM stages.

## CONCLUSION

This study provides the first evidence that LASt, derived from the ratio of transmitral flow velocity to atrial deformation (E:E’/εS), is significantly higher in cats with HCM. LASt values were about seven times higher in HCM cats than in normal cats (median 2.26 vs. 0.30), highlighting its sensitivity to atrial structural remodeling and reduced compliance. Similarly, strain parameters from STE (εS, εE, εA) were notably decreased, and several Doppler and TDI indices (E:A, MV A, MV E’, and MV S’) showed strong or moderate correlations with LASt, indicating that LASt reflects both atrioventricular functional interactions and diastolic load-dependent changes. Together, these findings establish LASt as a promising surrogate marker for detecting atrial dysfunction in feline HCM.

LASt provides a load-adjusted, noninvasive metric that combines atrial deformation with LV filling pressures, giving clinicians a more comprehensive assessment than strain alone. Its strong links to LA enlargement, decreased atrial pump function, and diastolic dysfunction suggest that LASt could improve early detection, assist in risk stratification, and potentially enhance monitoring of disease progression in cats with suspected or confirmed HCM. Since LA enlargement often occurs later in the disease, LASt’s ability to detect subclinical atrial remodeling may support earlier intervention and better prognostic planning.

A key strength is the first use of LASt in feline cardiology, supported by a consistent imaging protocol, standardized STE methodology, and single-operator acquisition to minimize variability. The study also offers species-specific correlations between LASt and various echocardiographic parameters, filling an important gap across human, canine, and feline cardiology.

Prospective, longitudinal studies with larger sample sizes are needed to validate LASt, establish clinically relevant diagnostic thresholds, and assess its prognostic value across various stages of HCM. Future research should explore whether LASt can predict outcomes such as CHF, thromboembolism, or death, and if it can help guide treatment decisions or monitor therapeutic responses. Comparative studies across different breeds and other cardiomyopathies would also enhance its clinical usefulness.

In summary, LASt represents a novel, sensitive, and physiologically integrated marker of atrial dysfunction in cats with HCM. Its significant elevation in diseased cats, along with strong correlations with structural and functional indices, highlights its potential as a valuable addition to routine echocardiographic assessment. With further validation, LASt could become an important clinical tool for detecting early atrial remodeling, improving diagnostic accuracy, and enhancing long-term management of feline HCM.

## DATA AVAILABILITY

The supplementary data can be made available from the corresponding author upon request.

## AUTHORS’ CONTRIBUTIONS

PT: Methodology, investigation, data curation, formal analysis, and drafting and editing the manuscript. JT: Methodology, investigation, and data curation. SDS: Conceptualization, investigation, data curation, formal analysis, visualization, supervision, and manuscript editing. All authors have read and approved the final version of the manuscript.
